# Bloodstream infection and pneumonia caused by *Chlamydia abortus* infection in China: a case report

**DOI:** 10.1186/s12879-022-07158-z

**Published:** 2022-02-23

**Authors:** Changjun Zhu, Minjie Lv, Jianling Huang, Changwen Zhang, Lixu Xie, Tianming Gao, Bo Han, Wenjing Wang, Ganzhu Feng

**Affiliations:** Department of Respiratory Medicine, the Second Affiliated Hospital of Nanjing Medical University, Nanjing, 210000 China

**Keywords:** Bloodstream infection, Pneumonia, *Chlamydia abortus*, Case report

## Abstract

**Background:**

*Chlamydia abortus* is generally considered to cause abortion, stillbirth, and gestational sepsis in pregnant women, but it’s rare in bloodstream infection and pneumonia.

**Case presentation:**

We present details of a patient with bloodstream infection and pneumonia caused by *Chlamydia abortus*. Both blood next-generation sequencing (NGS) and sputum NGS indicate *Chlamydia abortus* infection. The patient received intravenous infusion of piperacillin sodium and tazobactam sodium (4.5 g/8 h) and moxifloxacin (0.4 g/d) and oral oseltamivir (75 mg/day). Within one month of follow-up, the patient's clinical symptoms were significantly improved, and all laboratory parameters showed no marked abnormality. However, chest computer tomography (CT) showed the inflammation wasn’t completely absorbed. And we are still following up.

**Conclusions:**

*Chlamydia abortus* can cause pneumonia in humans. NGS has the particular advantage of quickly and accurately identifying the infection of such rare pathogens. Pneumonia is generally not life-threatening, and has a good prognosis with appropriate treatment. However, *Chlamydia* infection can lead to serious visceral complications which clinicians should pay attention to.

## Background

*Chlamydia* consisting of thirteen classified chlamydial species and three candidate species [1] which widely parasitizes mammals and birds and easily infects the mucosa. The *Chlamydia* infection leads to epidemiologically and clinically important diseases both in humans and animals [2], in which the most common pathogens are *Chlamydia psittaci*, *Chlamydia trachomatis*, and *Chlamydia pneumoniae*.

*Chlamydia abortus* is a kind of zoonotic pathogen, which has been reported to infect a variety of animals including goats, sheep, yaks, pigs, horses, rabbits, guinea pigs, mice, and farmed fur animals [3–9]. Besides, *Chlamydia abortus* is also the causative pathogen of abortion [10], stillbirth, gestational sepsis [11–13], and pelvic inflammatory disease in humans [14]. Nevertheless, only one case of pneumonia associated with *Chlamydia abortion* has been reported worldwide [15]. Here, we describe a case of pneumonia caused by *Chlamydia abortus*, including clinical characteristics, signs, laboratory examination, imaging performance, diagnosis, and therapy. As we know, this is the first report of pneumonia in humans caused by *Chlamydia abortus* in China.

## Case presentation

A 66-year-old man was admitted to hospital with a fever, generalized weakness, cough, wheezing, headache, dizziness, nausea, and vomiting, with a history of hepatic malignancy and three interventional surgeries.

**Fig. 1:**
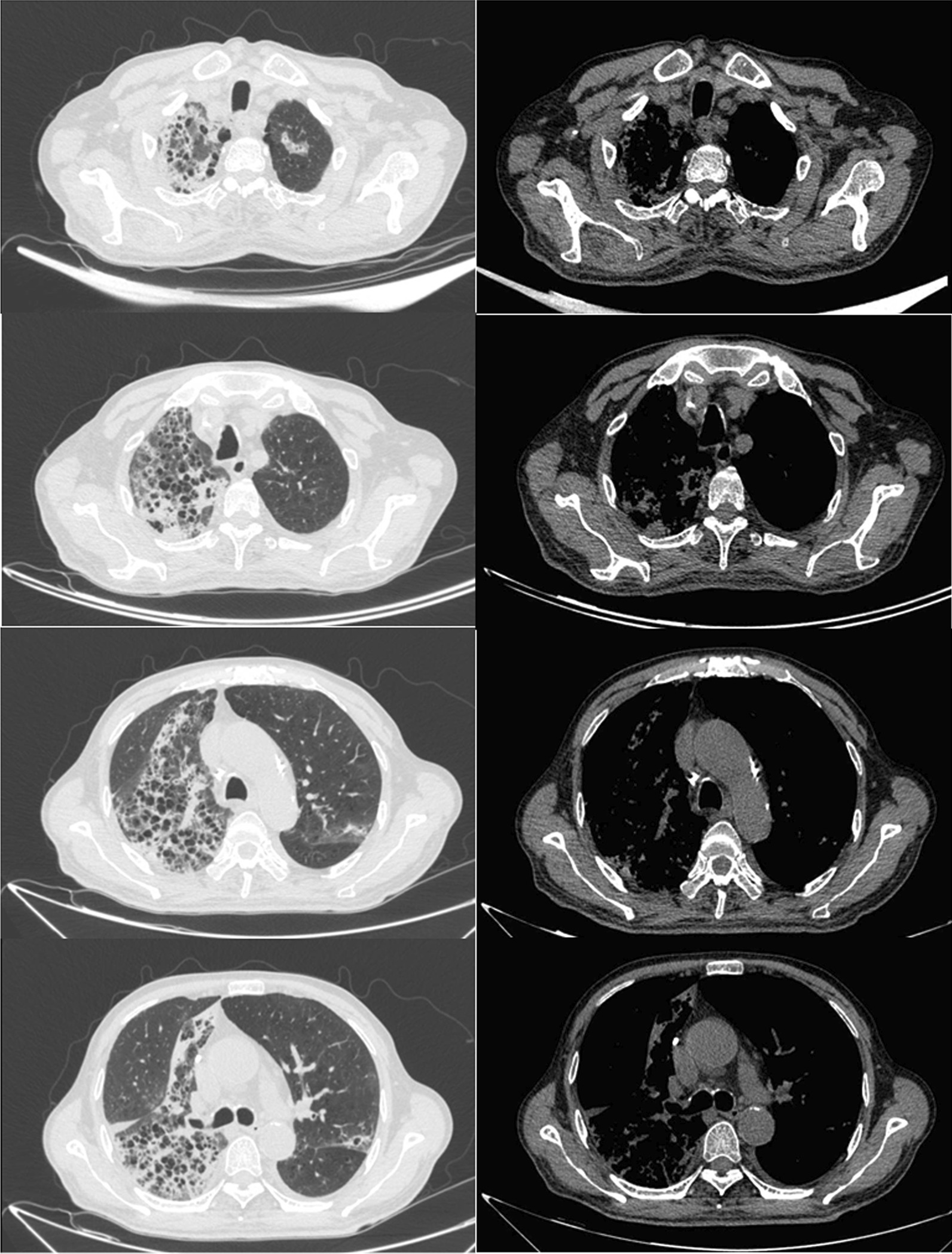
Multiple grid-shaped blurred shadows are seen in both lungs, especially in the right lung

On physical examination, the patient’s body temperature was 39.7 °C, and wet rales could be heard in the right lung. Relevant laboratory tests indicated type I respiratory failure, hyponatremia, pancytopenia, hypoproteinemia, elevated C-reactive protein (CRP), increased ferritin, and slightly elevated D-dimer, total bilirubin and glucose (Table 1). Arterial blood gas analysis (no oxygen inhalation) showed the following: pH 7.46; PaCO_2_ 37.2 mmHg; PaO_2_ 51.2 mmHg; blood lactate 2.3 mmol/L. Analysis of serology from multiple enzyme-linked immunosorbent assays was negative for IgM to legionella pneumophila serotype I, adenovirus, respiratory syncytial virus, mycoplasma pneumoniae antibody, influenza A, influenza B, and parainfluenza. No pathogenic bacteria were found in sputum and pharyngeal swab culture. There was no growth of aerobic and anaerobic bacteria in the blood culture for five days. The acid-fast staining of sputum was negative three times. The chest CT showed t multiple honeycomb changes in both lungs, especially the right lung (Fig. 1).

**Table 1:** Initial maternal laboratory results

Variable	Reference range	Result
Hemoglobin (g/dL)	12.0–16.0	9.8
White-cell count (per mm^3^)	4000–10,000	9780
Platelet count (per mm^3^)	100,000–300,000	116,000
Absolute lymphocyte count (per mm^3^)	800–4000	260
C-reactive protein (mg/dL)	0.00–1.00	16.07
Procalcitonin (ng/mL)	< 0.05	2.67
D-dimer (µg/mL)	0.00–1.00	4.05
Ferritin (ng/mL)	30–400	1242
CPK (U/L)	50.0–310.0	58
Glucose (mmol/L)	3.9–6.1	6.39
Creatinine (μmol/L)	57–111	65.3
Blood urea nitrogen (mmol/L)	3.6–9.5	7.58
Total bilirubin (μmol/l)	0.00–21.00	30.8
Aspartate aminotransferase (U/L)	15.0–40.0	53.4
Alanine aminotransferase (U/L)	9.0–50.0	29.7
Sodium (mmol/L)	137.0–147.0	131.1
Potassium (mmol/L)	3.5–5.3	4.87
Chloride (mmol/L)	99.0–110.0	95.4
Total protein(g/L)	65–85	48.7
Albumin(g/L)	40–55	24.9
Globulin(g/L)	20–40	23.8
Arterial blood gases		
pH	7.35–7.45	7.461
PCO_2_	35–45	37.2
PO_2_	83–108	51.2
HCO_3_	18.0–23.0	26.6
BE	− 2.0 to 3.0	2.7

The patient was diagnosed with pneumonia, type I respiratory failure, pleural effusion, hepatic malignancy, hyponatremia, hypochromic microcytic anemia, and hypoproteinemia on admission. Next, the patient was treated with intravenous infusion of piperacillin sodium and tazobactam sodium (4.5 g/8 h) and moxifloxacin (0.4 g/day) and oral oseltamivir (75 mg/day), supplemented with amino acids, fat emulsion, and gamma globulin, improving the symptom of respiratory failure via oxygen inhalation.

**Fig. 2:**
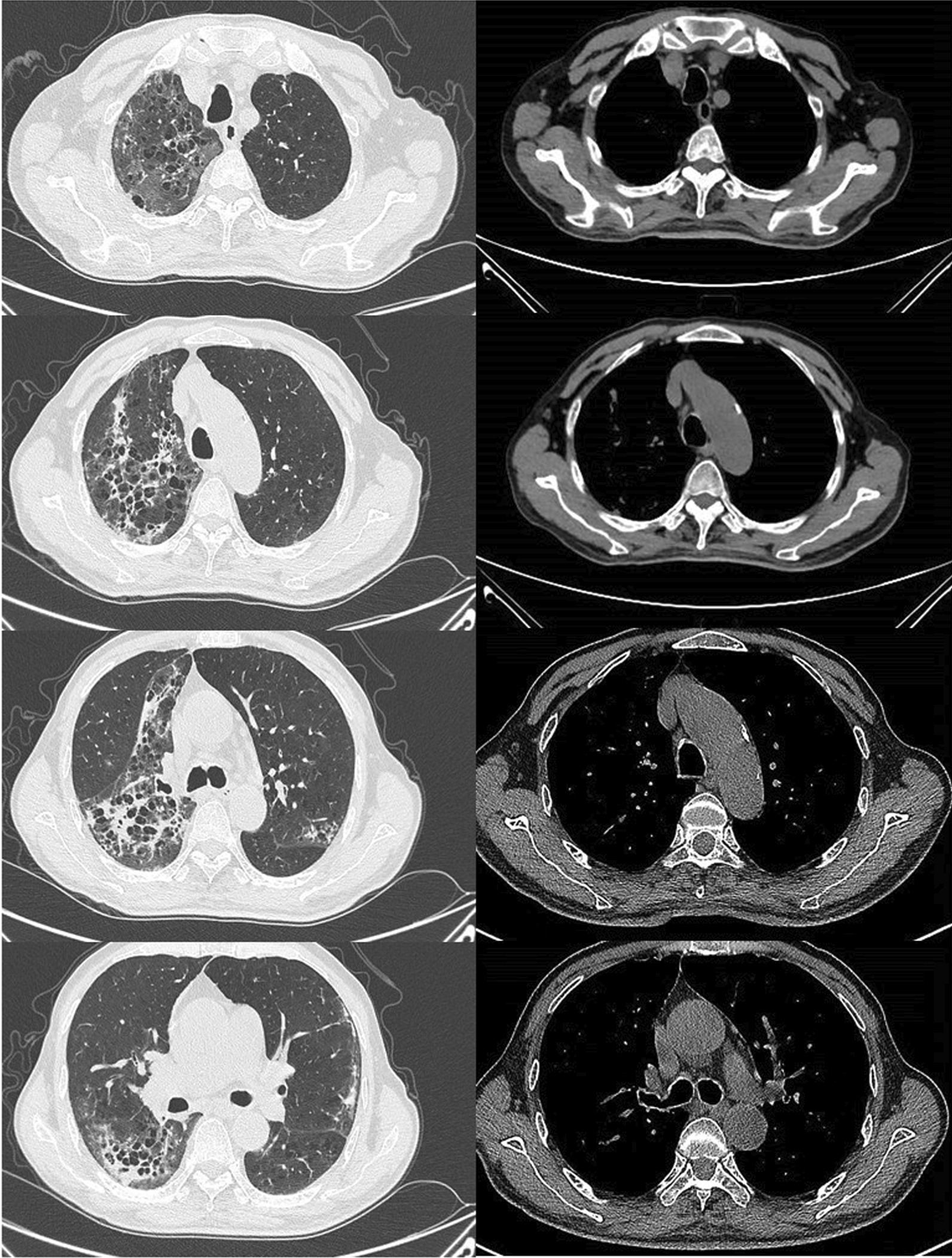
Multiple reticular blurred shadows in both lungs, bilateral pleural thickening

Although relevant laboratory tests and chest CT have been performed, no pathogen was found. The patient was in critical condition with continuous high fever and respiratory failure. We performed peripheral blood NGS and sputum NSG for pathogen detection on the second and sixth days of admission, respectively. The results of NGS suggested a high possibility of *Chlamydia abortus* (Tables 2, 3).Giving that *Chlamydia* infection is usually mixed infection, treatment was continued with piperacillin sodium and tazobactam sodium in combination with moxifloxacin [16]. Delightedly, the symptoms of the patient were improved significantly after three days. However, the patient developed a significant decrease in platelets and hemoglobin on the seventh day during the treatment (platelet counts 47*10^9^/L, hemoglobin 68 g/L, red blood cell counts 3.5*10^12^/L). It was probably caused by the infection, but hematological diseases could not be excluded. Subsequently, the patient's above-mentioned indicators were significantly improved after transfusing with 2u of suspended leukocyte-poor red blood cells. At the 10th day during the treatment, reexamined chest CT showed multiple reticular blurred shadows in both lungs, bilateral pleural thickening, and arc-shaped low-density shadows in both thoracic cavities, indicating interstitial pneumonia and bilateral pleural effusion (Fig. 2). At the 16th day during the treatment, high-sensitivity CRP (12.24 mg/L) and procalcitonin (0.231 ng/mL) were improved, but anemia and hypoproteinemia still existed, and arterial blood gases showed PO_2_ 56 mmHg and PO_2_ 43 mmHg.

**Table 2:** Next generation sequencing results in peripheral blood

Mycoplasma/Chlamydia/Rickettsia					
Genus			Species		
Name	Sequence number	Relative abundance %	Name	Sequence number	Relative abundance %
*Chlamydia*	754	98.18	*Chlamydia abortus*	618	80.47

**Table 3:** Next generation sequencing results in sputum

Mycoplasma/Chlamydia/Rickettsia					
Genus			Species		
Name	Sequence number	Relative abundance %	Name	Sequence number	Relative abundance %
*Chlamydia*	75	13.54	*Chlamydia abortus*	64	11.55

**Fig. 3:**
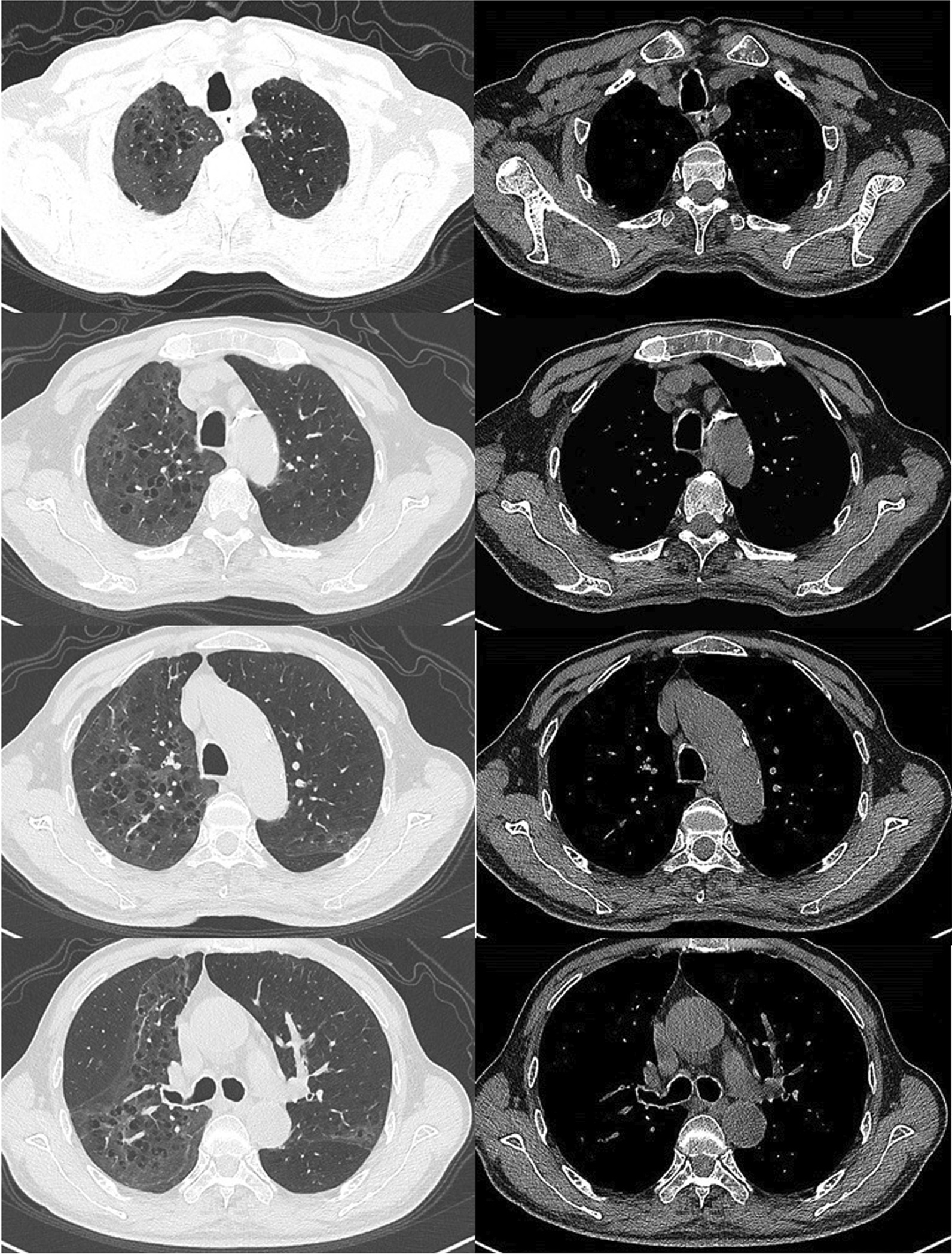
Multiple grid-shaped blurred shadows were observed in both lungs, which were obviously absorbed

Soon afterwards, the patient was transferred to the infectious disease hospital for further treatment with positive antibodies specific to hepatitis B and syphilis. After admission, the patient was treated with intravenous infusion of piperacillin sodium and tazobactam sodium (4.5 g/8 h) and moxifloxacin (0.4 g/day) for 16 days, with oral oseltamivir for 7 days. Subsequently, the patient was instructed to take moxifloxacin (0.4 g/day) and clarithromycin (0.25 g/12 h) within two weeks of discharge. The patient complained of no cold, fever, headache, dizziness, muscle soreness, cough, expectoration, chest distress or asthma. Laboratory tests showed white blood cell count 4.98*10^9^/L, hypersensitive CRP 7.94 mg/L, creatinine 66 μmol/L, alanine aminotransferase 31 U/L, and aspartate aminotransferase 26.1 U/L. Chest CT showed remarkable improvement of interstitial inflammation and emphysema in both lungs (Fig. 3).

## Discussion and conclusions

*Chlamydia abortus* infection is usually reported in pregnant women, beginning with influenza-like illness and progressing to thrombocytopenia and coagulation dysfunction, often leading to fetal death.

So far, only two cases of extra-gestational manifestations of *Chlamydia abortus* infection have been reported in humans [14, 15].

*Chlamydia abortus* is excreted through the urine, feces, milk, amniotic fluid, placenta, aborted fetus and other routes of sick animals. Most reported human infections result from direct contact between pregnant women and infected animals. Also, several cases describe indirect contact through visiting or living on or near farms affected by endemic abortion, which suggests that clinicians should particularly pay attention to people who have been in contact with animals from infected herds.

At present, the *chlamydia abortus* infection is mainly diagnosed by PCR according to literatures. In this case report, NGS clears the diagnosis of *chlamydia abortus* infection. The blood NGS result showed the patient's bloodstream infection caused by *chlamydia abortus*, but other pathogens could not be ruled out to cause pneumonia. Therefore, we performed NGS of sputum and confirmed that *chlamydia abortus* is the pathogen of pneumonia in this patient.

NGS, also known as high-throughput sequencing, which can perform sequencing for all nucleic acids in a sample [17, 18]. NGS is characterized by fast detection speed, high accuracy, low cost, wide coverage and huge output [19]. It can theoretically detect all pathogens of clinical samples and is especially suitable for atypical, rare, and new pathogens. In 2014, Wilson et al. applied NGS to detect *leptospirosis* in cerebrospinal fluid samples of children, which is the first application of NGS in the diagnosis of infectious diseases [20]. Subsequently, unbiased metagenomic next-generation sequencing (mNGS) has revolutionized our ability to discover emerging pathogens, especially newly identified viruses. Miao et al. reported the largest retrospective study on the detection of pathogens of infectious diseases by mNGS in China. The study included a total of 511 samples and reported the sensitivity and specificity of mNGS were 50.7% and 85.7%, respectively, which were significantly higher than that of traditional testing methods, especially in the detection of pathogens such as *Mycobacterium tuberculosis*, viruses, fungi, and anaerobic bacteria. Additionally, the result of mNGS is hardly affected by prior antibiotic exposure [21].

*Chlamydia* can cause serious visceral complications after systemic infection, which is worthy of the attention of clinicians [12, 22]. In this case report, the patient was immunocompromised due to the history of liver malignancy and exhibited symptoms of respiratory failure, pleural effusion, hyponatremia, hypochromic microcytic anemia and hypoproteinemia after *chlamydia abortus* infection, which urged clinicians to confirm the diagnosis as soon as possible and actively intervene. On the basis of literature [23], preferred moxifloxacin combined with piperacillin sodium and tazobactam sodium because the *chlamydia* infections were mostly mixed. The patient's clinical symptoms and inflammation indicators were significantly improved after two weeks of treatment. After discharge, the patient was instructed to continue on clarithromycin and moxifloxacin. A one-month follow-up showed that the patient's clinical symptoms were significantly improved and there was no significant abnormality in all laboratory parameters. But chest CT showed the inflammation was not completely absorbed, indicating imaging recovery of pneumonia may take several months. We are still following up.

## Data Availability

Not applicable.

## Abbreviations

NGS: Next-generation sequencing
CT: Computer tomography
CRP: C-reactive protein
mNGS: Metagames metagenomic next-generation sequencing

## References

[1] Bommana S, Polkinghorne A (2019). Mini review: Antimicrobial control of chlamydial infections in animals: current practices and issues. Front Microbiol.

[2] Longbottom D, Coulter LJ (2003). Animal chlamydioses and zoonotic implications.

[3] Li Z, Cao X, Fu B, Chao Y, Cai J, Zhou J (2015). Identification and Characterization of *Chlamydia abortus* isolates from Yaks in Qinghai, China. Biomed Res Int.

[4] Li Z, Liu P, Cao X, Lou Z, Zaręba-Marchewka K, Szymańska-Czerwińska M, Niemczuk K, Hu B, Bai X, Zhou J (2018). First report of *Chlamydia abortus* in farmed fur animals. Biomed Res Int.

[5] Campos-Hernández E, Vázquez-Chagoyán JC, Salem AZM, Saltijeral-Oaxaca JA, Escalante-Ochoa C, López-Heydeck SM, de Oca-Jiménez RM (2014). Prevalence and molecular identification of *Chlamydia abortus* in commercial dairy goat farms in a hot region in Mexico. Trop Anim Health Pro.

[6] Teankum K, Pospischil A, Janett F, Brugnera E, Hoelzle LE, Hoelzle K, Weilenmann R, Zimmermann DR, Gerber A, Polkinghorne A, Borel N (2007). Prevalence of chlamydiae in semen and genital tracts of bulls, rams and bucks. Theriogenology.

[7] Ruhl S, Goy G, Casson N, Thoma R, Pospischil A, Greub G, Borel N (2008). *Parachlamydia acanthamoebae* infection and abortion in small ruminants. Emerg Infect Dis.

[8] Salinas J, Ortega N, Borge C, Rangel MJ, Carbonero A, Perea A, Caro MR (2012). Abortion associated with *Chlamydia abortus* in extensively reared Iberian sows. Vet J.

[9] Szymańska-Czerwińska M, Mitura A, Zaręba K, Schnee C, Koncicki A, Niemczuk K (2017). Poultry in Poland as Chlamydiaceae carrier. J Vet Res.

[10] Pospischil A, Thoma R, Hilbe M, Grest P, Zimmermann D, Gebbers JO (2002). Abortion in humans caused by *Chlamydophila abortus* (Chlamydia psittaci serovar 1). Schweiz Arch Tierheilkd.

[11] Pichon N, Guindre L, Laroucau K, Cantaloube M, Nallatamby A, Parreau S (2020). *Chlamydia abortus* in pregnant woman with acute respiratory distress syndrome. Emerg Infect Dis.

[12] Walder G, Hotzel H, Brezinka C, Gritsch W, Tauber R, Würzner R, Ploner F (2005). An unusual cause of sepsis during pregnancy: recognizing infection with *Chlamydophila abortus*. Obst Gynecol.

[13] Roberts W, Grist NR, Giroud P (1967). Human abortion associated with infection by ovine abortion agent. BMJ.

[14] Walder G, Meusburger H, Hotzel H, Oehme A, Neunteufel W, Dierich MP, Würzner R (2003). *Chlamydophila abortus* pelvic inflammatory disease. Emerg Infect Dis.

[15] Ortega N, Caro MR, Gallego MC, Murcia-Belmonte A, Álvarez D, Del Río L, Cuello F, Buendía AJ, Salinas J (2015). Isolation of *Chlamydia abortus* from a laboratory worker diagnosed with atypical pneumonia. Irish Vet J.

[16] Kohlhoff SA, Hammerschlag MR (2015). Treatment of Chlamydial infections: 2014 update. Expert Opin Pharmacother.

[17] McCombie WR, McPherson JD, Mardis ER (2019). Next-generation sequencing technologies. Cold Spring Harb Perspect Med.

[18] Lecuit M, Eloit M (2015). The potential of whole genome NGS for infectious disease diagnosis. Expert Rev Mol Diagn.

[19] Steuernagel B, Taudien S, Gundlach H, Seidel M, Ariyadasa R, Schulte D, Petzold A, Felder M, Graner A, Scholz U, Mayer KFX, Platzer M, Stein N (2009). De novo 454 sequencing of barcoded BAC pools for comprehensive gene survey and genome analysis in the complex genome of barley. BMC Genomics.

[20] Wilson MR, Naccache SN, Samayoa E, Biagtan M, Bashir H, Yu G, Salamat SM, Somasekar S, Federman S, Miller S, Sokolic R, Garabedian E, Candotti F, Buckley RH, Reed KD, Meyer TL, Seroogy CM, Galloway R, Henderson SL, Gern JE, DeRisi JL, Chiu CY (2014). Actionable diagnosis of neuroleptospirosis by next-generation sequencing. N Engl J Med.

[21] Miao Q, Ma Y, Wang Q, Pan J, Zhang Y, Jin W, Yao Y, Su Y, Huang Y, Wang M, Li B, Li H, Zhou C, Li C, Ye M, Xu X, Li Y, Hu B (2018). Microbiological diagnostic performance of metagenomic next-generation sequencing when applied to clinical practice. Clin Infect Dis.

[22] Rohde G, Straube E, Essig A, Reinhold P, Sachse K (2010). Chlamydial zoonoses. DTSCH Arztebl Int.

[23] de Barbeyrac B, Bébéar C (2005). Histoire naturelle des infections à Chlamydiaphysiopathologie des infections à Chlamydia: Conséquences diagnostiques et thérapeutiques. Arch Pediatr.

